# Dual-Activated Tamarix Gallica-Derived Carbons for Enhanced Glyphosate Adsorption: A Comparative Study of Phosphoric and Sulfuric Acid Activation

**DOI:** 10.3390/ma18030511

**Published:** 2025-01-23

**Authors:** Saliha Benaoune, Abdelkarim Merzougui, Rania Remmani, Narimene Bouzidi, Antonio Ruiz-Canales, Imane Akacha, Amir Djellouli

**Affiliations:** 1 Research Laboratory in Civil Engineering, Hydraulics, Sustainable Development and Environment (LAR-GHYDE), University Mohamed Khider, Biskra 07000, Algeria; saliha.benaoune@univ-biskra.dz (S.B.); a.merzougui@univ-biskra.dz (A.M.); imane.akacha@univ-biskra.dz (I.A.); 2 Department of Engineering, Miguel Hernández University, 03312 Alicante, Spain; aruizcanales@gmail.com; 3 Scientific and Technical Research Center on Arid Regions Omar El-Bernaoui, Biskra 07000, Algeria; bznarimene@yahoo.fr (N.B.); amir.djellouli@yahoo.fr (A.D.)

**Keywords:** glyphosate adsorption, Tamarix gallica, activated carbon, chemical activation, water treatment, adsorption isotherms

## Abstract

This study investigates the efficacy of activated carbons (ACs) derived from Tamarix gallica (TG) leaves for glyphosate removal from aqueous solutions. Two chemical activation methods, using phosphoric acid (H_3_PO_4_) and sulfuric acid (H_2_SO_4_), were compared to optimize adsorbent performance. The resulting materials, labeled AC-H_3_PO_4_ and AC-H_2_SO_4_, were comprehensively characterized using XRD, FTIR, SEM-EDS, BET analysis, and pHpzc determination, revealing distinct physicochemical properties. AC-H_3_PO_4_ exhibited a larger surface area (580.37 m^2^/g) and more developed pore structure compared to AC-H_2_SO_4_ (241.58 m^2^/g). Adsorption kinetics were best described by the pseudo-first-order model for both adsorbents. Isothermal studies demonstrated that AC-H_3_PO_4_ followed a pore-filling mechanism best described by the Dubinin–Radushkevich model, while AC-H_2_SO_4_ showed multilayer adsorption fitting the Freundlich model. Both adsorbents exhibited high glyphosate removal capacities, with maximum Langmuir adsorption capacities of 247.58 mg/g and 235.13 mg/g for AC-H_3_PO_4_ and AC-H_2_SO_4_, respectively. The mean free energy of adsorption (E) values confirmed physisorption as the dominant mechanism. This research highlights the potential of TG-derived activated carbons as sustainable and effective adsorbents for glyphosate remediation in water treatment applications, demonstrating the impact of activation methods on adsorption performance.

## 1. Introduction

Herbicide pollution, particularly from glyphosate, is a growing environmental concern due to its extensive agricultural use and persistence in aquatic ecosystems [1]. Glyphosate poses significant risks to both environmental and human health as it infiltrates water sources, causing long-term ecological damage [2]. Conventional water treatment technologies, such as chemical coagulation and activated sludge, often fail to efficiently or economically remove glyphosate, especially in resource-limited settings [3,4]. Adsorption using AC has emerged as an effective technique for removing such contaminants due to its high surface area and porosity, making it a valuable tool for water purification [5]. However, the production of AC from non-renewable sources, such as coal, is unsustainable and energy-intensive, prompting the search for eco-friendly alternatives [6].

In this context, biomass-derived AC has garnered significant attention as a sustainable and cost-effective solution for water treatment [7]. This study investigates the potential of TG, a halophytic plant that thrives in saline and arid conditions, as a biomass precursor for AC production. TG offers several environmental advantages, including its ability to grow in regions facing water scarcity, such as Algeria, and its low cultivation costs due to its resilience in harsh conditions [8]. The biomass of TG is characterized by a substantial content of lignin and cellulose, which play a critical role in the structural integrity and porosity development of the resulting AC. Lignin, a complex aromatic polymer, contributes to the rigidity and stability of the biomass, while cellulose, a polysaccharide, provides a fibrous structure that enhances the textural properties of the AC. During the activation process, lignin aids in maintaining the structural framework, while cellulose undergoes decomposition and crosslinking, leading to the formation of a porous network. By utilizing TG, this study aligns with the global push towards sustainable water treatment solutions, providing a renewable and locally available alternative to conventional AC [8]. This research not only addresses the environmental challenge of glyphosate pollution but also contributes to the circular economy by promoting the use of renewable biomass.

The adsorption efficiency of biomass-derived AC is largely dependent on its activation process. In this study, TG biomass is chemically activated using a dual activation method with phosphoric and sulfuric acids, which enhances its porosity and surface area [9]. This dual activation process is crucial for increasing the number of active sites on the AC, thereby improving its adsorption capacity for glyphosate. The chemical treatment optimizes the surface functionality of TG-AC, facilitating various interactions with glyphosate, such as hydrogen bonding, electrostatic attraction, and van der Waals forces [10]. This research aims to improve the adsorption performance of TG-derived AC, offering a more sustainable alternative to conventional adsorbents in water treatment.

Despite significant advancements in adsorption technologies, several critical research gaps persist, particularly in herbicide removal from water. Current adsorbents are frequently expensive or derived from non-renewable resources, making them less viable for widespread environmental applications. Biomass-derived adsorbents, such as those synthesized from TG, represent a promising and sustainable alternative. TG, a halophytic plant from the Tamaricaceae family, thrives in arid climates with high salinity, making it naturally abundant in regions like Algeria. Its high lignin and cellulose content not only ensures structural integrity during activation but also enhances its adsorption potential. Additionally, TG is cost-effective and environmentally friendly due to its natural abundance and resilience in harsh conditions [11]. This study introduces a novel approach by employing TG as a precursor for AC synthesis, utilizing a dual-chemical activation process with phosphoric and sulfuric acids. This dual activation method significantly enhances the material’s porosity and surface area, optimizing its adsorption capacity for glyphosate. Furthermore, while adsorption studies often focus solely on either isotherms or kinetics [12], this research integrates both, providing a comprehensive evaluation of the adsorption mechanisms [13]. By addressing these gaps, this study contributes to the development of cost-effective and sustainable adsorbents for herbicide remediation in water treatment applications.

## 2. Materials and Methods

### 2.1. Biomass Precursor and Chemical Reagents

The biomass precursor for this study, Tamarix gallica (*French tamarisk*), was harvested from the Djemorah district of Biskra city (34°51′ N, 5°44′ E), Algeria, during the spring season (March–May). This region’s arid climate, characterized by low annual rainfall (120 mm) and wide temperature fluctuations (12 °C to 40 °C), contributes to the plant’s unique physiological adaptations, making it an ideal candidate for adsorbent production [14,15].

The harvested TG leaves underwent a systematic pre-treatment process to optimize their quality for activated carbon production. Initially, the leaves were air-dried for five days at ambient temperature to reduce moisture content while preserving cellular structure. Subsequently, the dried leaves were subjected to multiple washing cycles with deionized water to remove surface impurities and water-soluble compounds. The washed material was then oven-dried at 110 °C for 24 h (Memmert, Schwabach, Germany) to achieve a constant weight. Finally, the dried leaves were ground using a high-speed rotary mill and sieved to obtain a particle size range of 0.08–0.25 mm, optimizing surface area for the subsequent activation process [16,17].

All chemical reagents used in this study were of analytical grade and employed without further purification. The activation process utilized phosphoric acid (H_3_PO_4_, 85%, Biochem Chemopharma, Loire, France) and sulfuric acid (H_2_SO_4_, 98%, Biochem Chemopharma). These activating agents were selected for their proven efficiency in creating well-developed porous structures in lignocellulosic materials [18,19]. Additional reagents included hydrochloric acid (HCl, Biochem Chemopharma, Loire, France) and sodium hydroxide (NaOH, Biochem Chemopharma, Loire, France) for pH adjustments. The target adsorbate, N-(phosphonomethyl)glycine (Glyphosate, C_3_H_8_NO_5_P, 96%, Sigma-Aldrich, St. Louis, MO, USA), was selected due to its widespread use as a herbicide and growing environmental concerns [20,21].

### 2.2. Activated Carbons Synthesis

The preparation of ACs from TG leaves followed a two-stage process involving chemical pre-treatment and thermal activation. This method was selected for its efficiency in producing high-surface-area carbons with well-developed porosity, which is essential for effective adsorption [14,15].

The chemical activation procedure involved the separate impregnation of the prepared TG powder with either concentrated H_3_PO_4_ or H_2_SO_4_. The acids were not used as a mixture but were applied independently to ensure a clear understanding of their individual effects on the activation process. The biomass-to-acid ratio was maintained at 1:3 (*w*/*v*), as determined through preliminary experiments to optimize the surface area and pore volume of the resulting activated carbon [18]. The impregnation was carried out at room temperature under constant stirring to ensure uniform distribution of the activating agents throughout the biomass matrix.

Sulfuric acid played a critical role in the activation process by facilitating dehydration condensation reactions between hydroxyl groups in cellulose and lignin. This reaction enhances the stability and carbonization of the precursor material, leading to the formation of cross-links and a stable carbon framework. Phosphoric acid, on the other hand, contributed to the development of micropores and mesopores while introducing oxygen-containing functional groups, such as phosphates, which enhance the hydrophilicity and adsorption capacity of the activated carbon. The distinct roles of these acids were studied separately to better understand their individual contributions to the structural and chemical properties of the final product.

Following impregnation, the mixture was dried in an oven at 100 °C for 24 h. This drying step is crucial for initiating the chemical reactions between the activating agents and the organic components of the biomass, leading to the formation of cross-links and the development of a porous structure [19,20]. The final activation step involved heating the pre-treated char in a muffle furnace at 550 °C for 3 h under an oxygen-restricted atmosphere. This temperature and duration were optimized to maximize the development of porosity while minimizing excessive carbon burn-off [21]. After thermal activation, the resulting material was cooled to room temperature. The activated carbon was then subjected to a thorough washing process using hot deionized water to remove any residual chemicals and activation by-products. Washing continued until the pH of the filtrate reached neutrality. The washed activated carbon was dried overnight at 105 °C, cooled in a desiccator, and stored in airtight containers for further use.

### 2.3. Characterization of Activated Carbons

XRD analysis was performed to investigate the crystalline structure of the activated carbons. The diffraction patterns were obtained using a Bruker D8 Advance diffractometer equipped (Bruker, Karlsruhe, Germany) with CuKα radiation (λ = 1.54184 Å), operating at 40 kV and 40 mA. The XRD patterns were recorded in the 2θ range of 5° to 90°. FTIR spectroscopy was employed to identify the functional groups present on the surface of the activated carbons. The spectra were recorded using an Agilent Technologies Cary 630 spectrophotometer (Santa Clara, CA, USA) in the range of 400–4000 cm^−1^; samples were prepared using the KBr pellet method. This technique is vital for understanding the surface chemistry of ACs, which plays a significant role in their adsorption behavior. The surface morphology and textural properties of ACs were examined using SEM. EDS was conducted in conjunction with SEM to determine the elemental composition of the activated carbons. Nitrogen adsorption–desorption isotherms were measured at 77 K using a surface area analyzer to determine the textural properties of ACs. The specific surface area was calculated using BET method, while the pore size distribution was determined using the Barrett–Joyner–Halenda (BJH) method. The total pore volume was estimated from the amount of nitrogen adsorbed at a relative pressure (P/P0) of 0.99. The pH at pHpzc of ACs was determined using the pH drift method. The pHpzc was determined as the point where the final pH equals the initial pH [22].

### 2.4. Glyphosate Adsorption Experiments

A stock solution of GLY was prepared by dissolving the herbicide in deionized water. From this stock, working solutions of varying concentrations were created to meet the requirements of the adsorption experiments. Adsorption tests were carried out using AC as the adsorbent, with initial GLY concentrations ranging from 5 to 50 mg/L. All experiments were conducted at a temperature of 293 K, with the solution pH adjusted to 5. In each experiment, 40 mL of GLY solution at a specific concentration was placed in a 100 mL beaker and mixed with 10 mg of activated carbon. The solution was stirred continuously at 300 rpm for the duration required to reach adsorption equilibrium. After equilibrium was attained, the adsorbent–adsorbate mixture was filtered using a 0.45 μm membrane filter to separate the activated carbon from the solution. The residual GLY concentration in the filtrate was then analyzed using a DR 5000 UV-Vis spectrophotometer (Hach, Loveland, CO, USA) at a maximum absorption wavelength of 265 nm. The equilibrium adsorption capacity of the adsorbents, Qe (mg/g), was determined using the mass balance equation shown below:

*Qe* = (*C*_0_ − *C_e_*) × *V*/*m* (1)

where: C_0_ and C*e* represent the initial and equilibrium concentrations of glyphosate (mg/L), respectively, V is the volume of the glyphosate solution (L), and m is the mass of the activated carbon used (g).

Similarly, the adsorption capacity at any given time t, Qt (mg/g), was calculated using the same equation:

*Qt* = (*C*_0_ − *C_T_*) × *V*/*m* (2)

The removal efficiency of glyphosate was calculated as follows:

%Removal Efficiency = (*C*_0_ − *Ce*) × 100/*c*_0_ (3)

These equations were used to evaluate the adsorption capacity and removal efficiency of the activated carbon adsorbents for glyphosate under varying experimental conditions [15,16].

### 2.5. Modeling of the Adsorption Process

The experimentally obtained data were analyzed using four well-established isotherm models: Langmuir, Freundlich, Temkin, and Dubinin–Radushkevich (D-R) isotherms [17]. The Langmuir isotherm model assumes monolayer adsorption on a surface with identical adsorption sites, while the Freundlich model accounts for adsorption on heterogeneous surfaces with varying adsorption energies. The Langmuir model is expressed in its linearized form as follows:

Ce/qe = Ce/Qmax + 1/(K_L_ × Qmax) (4)

where: Qmax (mg/g) is the maximum adsorption capacity, and K_L_ is the Langmuir equilibrium adsorption constant [18].

The Freundlich isotherm, which describes adsorption on a heterogeneous surface, is linearized as follows:

Ln q_e_ = Ln K_F_ + (1/n) × Ln Ce (5)

where: K_F_ is the Freundlich adsorption constant, and n is the heterogeneity factor [19].

The Temkin and Dubinin–Radushkevich (D-R) isotherms, which account for adsorbate–adsorbent interactions and the porosity of the adsorbent, are given by the following equations, respectively:

qe = RT/b Ln (A × Ce)(6)

q_e_ = q_m_ × exp(−B_D_ [RTLn (1 + 1/Ce)]^2^) (7)

where: q_m_ is the theoretical monolayer adsorption capacity (mg/g), A is the Temkin constant, b is the heat of adsorption (J), B_D_ is the D-R constant related to the adsorption energy, and R is the universal gas constant (8.314 J/K·mol) [20].

To further understand the adsorption kinetics, pseudo-first-order (PFO) and pseudo-second-order (PSO) models were applied to the experimental data. These kinetic models are expressed as follows:

For PFO model: q_t_ = q_e_ (1 − exp(k_1_ × t)) (8)

For PSO model: q_t_ = (q_e_^2^ × k_2_ × t)/(1 + k_2_ × q_e_ × t) (9)

where: k_1_ and k_2_ are the rate constants for the PFO and PSO models, respectively [21].

To evaluate the accuracy of the adsorption models, both the coefficient of determination (R^2^) and the chi-square (χ^2^) test were employed:

(10) $$R^{2}=1-\frac{\sum_{i=1}^{N}{\left(q_{e,exp}-q_{e,cal}\right)}^{2}}{\sum_{i=1}^{N}{\left(q_{e,exp}-q_{e,mean}\right)}^{2}}$$

(11) $$\chi^{2}=\sum_{i=1}^{N}\frac{{\left(q_{e,exp}-q_{e,cal}\right)}^{2}}{q_{e,cal}}$$

where q_e,exp_ and q_e,cal_ are the experimental and calculated adsorption capacities, respectively. A lower χ^2^ value and an R^2^ value closer to 1 indicate a better fit of the model to the experimental data [23].

## 3. Results and Discussion

### 3.1. Characterization of Activated Carbons

**Figure 1:**
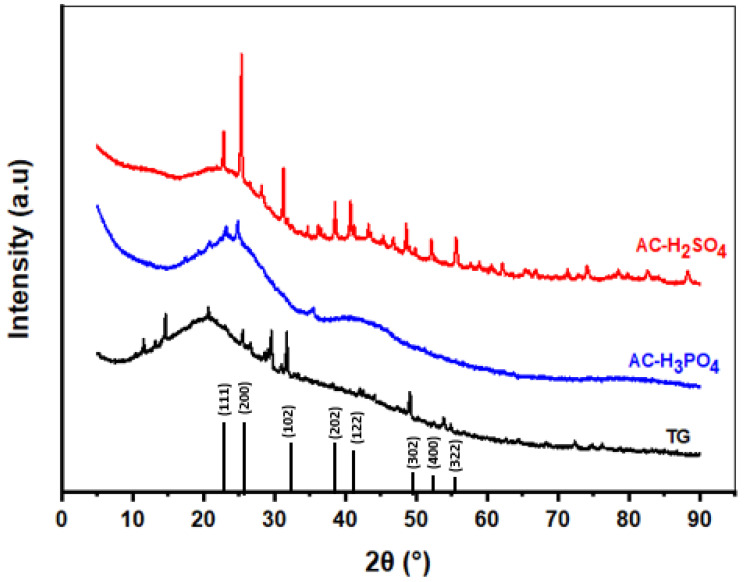
XRD patterns of AC-H_3_PO_4_, AC-H_2_SO_4_, and TG.

XRD patterns of TG, AC-H_3_PO_4_, and AC-H_2_SO_4_, as depicted in Figure 1, exhibit distinct characteristics that provide insights into the crystalline or amorphous nature of the materials. The XRD pattern of TG shows broad peaks, suggesting an amorphous structure, which is typical for lignocellulosic materials due to their disordered arrangement of cellulose, hemicellulose, and lignin [24]. After chemical activation with H_3_PO_4_, the XRD pattern of AC-H_3_PO_4_ retains a similar profile to TG, indicating that the activation process did not significantly alter the crystalline structure. This suggests that phosphoric acid activation primarily contributed to the formation of micropores and mesopores without inducing crystallization, maintaining the adsorbent’s amorphous nature [24]. The broad peaks are indicative of low crystallinity and small particle sizes, which is consistent with highly porous materials designed for adsorption applications [25].

In contrast, the XRD pattern of AC-H_2_SO_4_ reveals more distinct and sharper peaks at 2θ values of 22.94°, 25.2°, 31.58°, 38.67°, 40.92°, 48.71°, 52.01°, and 55.63°. These peaks indicate the presence of graphite-like structures and crystalline carbon domains, suggesting that sulfuric acid activation promotes a higher degree of structural order in the carbon material. The increased crystallinity can be attributed to the strong oxidizing nature of H_2_SO_4_, which facilitates localized rearrangements in the carbon matrix, leading to the development of ordered domains. This enhanced structural order may improve the material’s mechanical stability and provide a more structured surface conducive to adsorbate interactions [25].

The sharper diffraction peaks observed for AC-H_2_SO_4_, as compared to TG and AC-H_3_PO_4_, further indicate that H_2_SO_4_ activation induces greater order in the carbon atoms, while simultaneously expanding the pore structure. By contrast, the XRD pattern of AC-H_3_PO_4_ exhibits broader and less distinct peaks, reflecting a disordered, predominantly amorphous structure. This difference arises from the activation mechanisms: H_3_PO_4_ promotes cross-linking and the formation of micropores through its less reactive phosphate anion, whereas H_2_SO_4_ induces more significant oxidation and structural reorganization via its smaller and more reactive sulfate anion.

These structural variations significantly influence the adsorption performance of the materials. The predominantly amorphous structure of AC-H_3_PO_4_, characterized by a higher surface area and more developed porosity, is advantageous for accommodating a broad range of adsorbate molecules. Conversely, the increased crystallinity of AC-H_2_SO_4_ provides enhanced chemical stability and potentially stronger adsorbate–adsorbent interactions. This revised understanding underscores the critical role of activation agents in tailoring the microstructure of activated carbons for specific applications.

**Figure 2:**
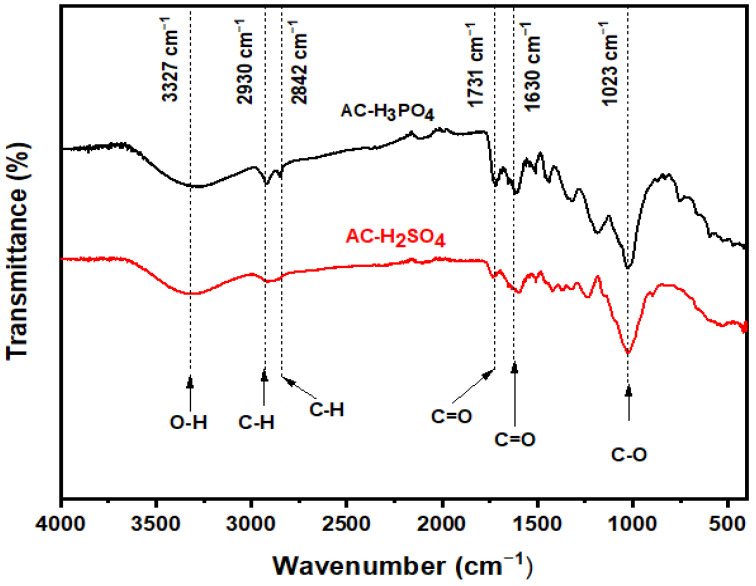
FTIR spectra of AC-H_3_PO_4_ and AC-H_2_SO_4_.

The FTIR spectra of AC-H_3_PO_4_ and AC-H_2_SO_4_, shown in Figure 2, provide key information about the surface functional groups of the activated carbons. The broad band at 3327 cm^−1^ in both spectra corresponds to the O-H stretching vibrations, which could be attributed to hydroxyl groups such as those in alcohols, phenols, or carboxyls, as well as adsorbed water molecules [12]. This indicates the presence of hydroxyl functionalities on the surface, contributing to the hydrophilicity of the adsorbents. The peaks at 2930 cm^−1^ and 2842 cm^−1^, observed in both spectra, are associated with the C-H stretching vibrations of aliphatic chains, representing asymmetrical and symmetrical elongations, respectively [26]. These C-H bonds suggest that the carbon materials contain aliphatic hydrocarbon chains, a typical feature of activated carbons. At 1731 cm^−1^, a weaker peak can be seen, which is attributed to the C=O stretching vibrations from carbonyl groups such as aldehydes, ketones, and esters, formed during the thermal degradation of cellulose and hemicellulose [27]. This peak is more pronounced in AC-H_3_PO_4_, which may suggest a higher content of oxygen-containing functional groups on its surface. Furthermore, the peak at 1630 cm^−1^ corresponds to the stretching vibrations of C=C bonds in aromatic rings, indicative of the graphitic carbon backbone of the activated carbons [28]. This is slightly more intense in AC-H_2_SO_4_, likely reflecting a more ordered carbon structure after sulfuric acid activation. Lastly, the band around 1023 cm^−1^ corresponds to the C-O stretching vibrations, possibly indicating the presence of ether or ester groups [28]. Overall, the FTIR spectra of both AC-H_3_PO_4_ and AC-H_2_SO_4_ show common functional groups, but the slight differences in peak intensities suggest variations in the surface chemistry due to the different activation agents. The presence of oxygen-containing groups such as O-H, C=O, and C-O is crucial for enhancing the adsorption capacity of these activated carbons, as they can participate in electrostatic interactions and hydrogen bonding with adsorbates such as pollutants.

**Figure 3:**
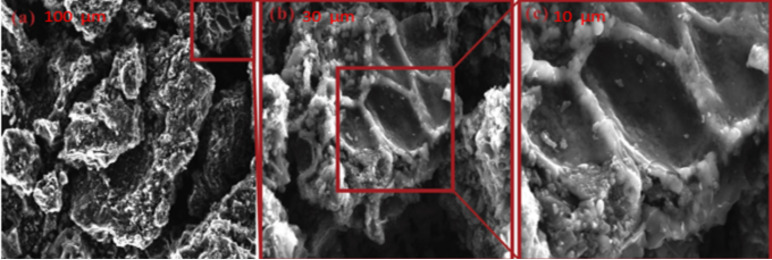
SEM micrographs of AC-H_3_PO_4_ sample at increasing magnifications ((**a**) 100 μm, (**b**) 30 μm, and (**c**) 10 μm).

**Figure 4:**
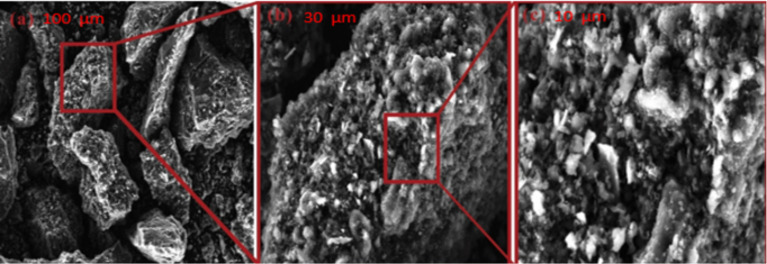
SEM micrographs of AC-H_2_SO_4_ sample at increasing magnifications ((**a**) 100 μm, (**b**) 30 μm, and (**c**) 10 μm).

SEM micrographs demonstrate pronounced morphological differences between the activated carbon samples treated with phosphoric acid (AC-H_3_PO_4_; Figure 3) and sulfuric acid (AC-H_2_SO_4_; Figure 4), elucidating the impact of activation agents on pore development. AC-H_3_PO_4_ exhibits a heterogeneous surface characterized by a hierarchical pore network, encompassing interconnected macro-, meso-, and micropores. This intricate structure, resembling a labyrinthine architecture, suggests enhanced accessibility for adsorbate molecules and potentially superior mass transfer properties. Conversely, AC-H_2_SO_4_ presents a more uniform surface topography with a predominance of smaller, more evenly distributed pores. The smoother texture and apparent surface etching in AC-H_2_SO_4_ indicate a distinct activation mechanism, possibly resulting in a higher density of adsorption sites but potentially limited pore interconnectivity. These morphological variations between AC-H_3_PO_4_ and AC-H_2_SO_4_ are likely to engender divergent adsorption behaviors, with AC-H_3_PO_4′_s diverse pore spectrum potentially favoring the capture of a broader range of molecular sizes, while AC-H_2_SO_4′_s homogeneous structure may excel in the adsorption of smaller molecules or offer enhanced surface area for interaction. The observed structural differences underscore the critical role of activation agent selection in tailoring carbon adsorbents for specific environmental remediation applications, particularly in the context of glyphosate removal from aqueous solutions.

EDS analysis reveals distinct elemental compositions for AC-H_3_PO_4_ and AC-H_2_SO_4_, elucidating the impact of activation agents on the AC chemical characteristics. AC-H_3_PO_4_ exhibits a higher carbon content (60.15% by weight) compared to AC-H_2_SO_4_ (58.36%), suggesting a more extensive carbonization process during phosphoric acid activation. Notably, AC-H_3_PO_4_ demonstrates elevated nitrogen content (16.32% vs. 12.22% in AC-H_2_SO_4_), which may enhance its adsorption capacity through increased basic functional groups, analogous to observations in other phosphoric acid-activated biomass-derived carbons [29]. The oxygen content, slightly higher in AC-H_3_PO_4_ (22.11%) than in AC-H_2_SO_4_ (20.56%), indicates a greater presence of oxygen-containing surface functionalities, potentially contributing to improved hydrophilicity and diverse adsorption mechanisms. The compositional variations between AC-H_3_PO_4_ and AC-H_2_SO_4_ likely stem from the distinct chemical interactions during activation, with phosphoric acid promoting crosslinking and structural expansion, while sulfuric acid may induce more pronounced surface etching and oxidation [30]. These elemental differences are expected to influence the AC surface chemistry, affecting their respective affinities for glyphosate and other contaminants in aqueous solutions. The higher nitrogen and oxygen contents in AC-H_3_PO_4_ suggest a potentially greater capacity for electrostatic interactions and hydrogen bonding, which could be advantageous for glyphosate adsorption, whereas the unique elemental profile of AC-H_2_SO_4_ may confer specific adsorption characteristics that warrant further investigation in targeted environmental remediation applications.

The textural properties of ACs, as elucidated by BET analysis, reveal striking disparities between H_3_PO_4_ and H_2_SO_4_ activation methods. AC-H_3_PO_4_ demonstrates superior porosity development, evidenced by its substantially higher BET surface area (580.37 m^2^/g) compared to AC-H_2_SO_4_ (241.58 m^2^/g). This disparity extends to Langmuir surface areas, with AC-H_3_PO_4_ exhibiting 1107.40 m^2^/g versus 416.32 m^2^/g for AC-H_2_SO_4_. T-Plot analysis further corroborates these findings, showing AC-H_3_PO_4_ possesses larger micropore (379.73 m^2^/g) and external surface areas (200.64 m^2^/g) compared to AC-H_2_SO_4_ (192.80 m^2^/g and 48.78 m^2^/g, respectively). This suggests a more intricate pore network in the AC-H_3_PO_4_. Total pore volume measurements align with these observations, as AC-H_3_PO_4_ displays higher values (0.38 cm^3^/g adsorption, 0.43 cm^3^/g desorption) than AC-H_2_SO_4_ (0.14 cm³/g adsorption, 0.16 cm³/g desorption). Notably, BJH analysis reveals comparable average pore diameters for both ACs (9.49–10.08 nm for AC-H_3_PO_4_; 10.32–10.78 nm for AC-H_2_SO_4_), placing them predominantly in the mesoporous category. However, a significant difference in average particle size is observed, with AC-H_3_PO_4_ exhibiting smaller particles (10.34 nm) compared to AC-H_2_SO_4_ (24.84 nm).

**Figure 5:**
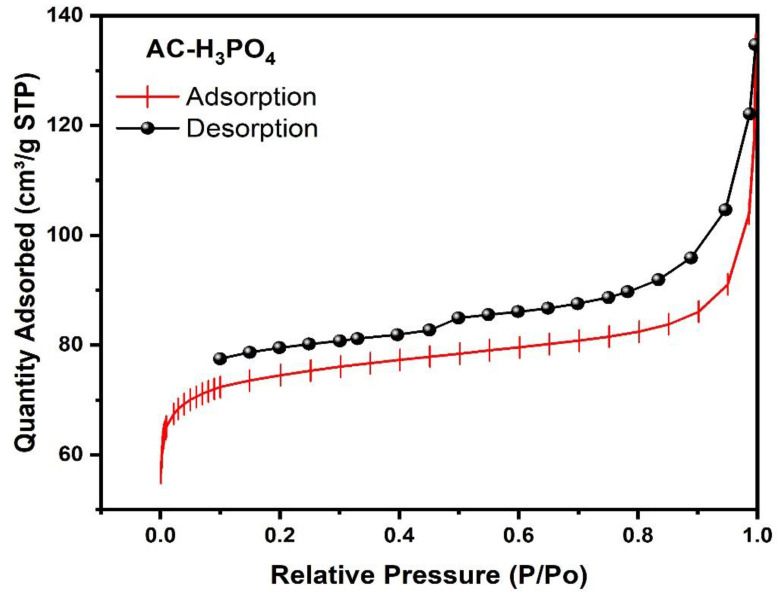
Nitrogen adsorption–desorption isotherm of AC-H_3_PO_4_ sample.

**Figure 6:**
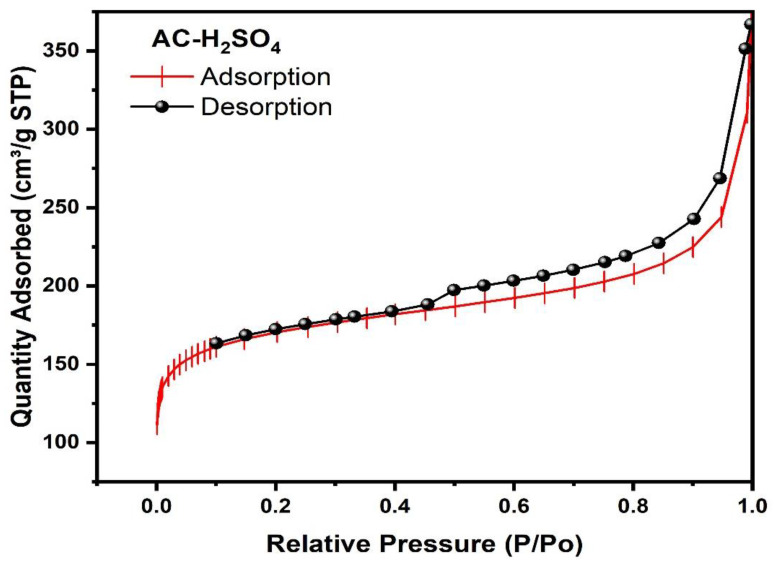
Nitrogen adsorption–desorption isotherm of AC-H_2_SO_4_ sample.

Nitrogen adsorption–desorption isotherms (Figure 5 and Figure 6) provide critical insights into the porous structures of ACs derived from Tamarix gallica. Both AC-H_3_PO_4_ and AC-H_2_SO_4_ exhibit Type IV isotherms, as classified by IUPAC, which are characteristic of mesoporous materials. This classification is consistent with the presence of capillary condensation within mesopores, a phenomenon that is further evidenced by the observed hysteresis loops. AC-H_3_PO_4_ demonstrates significantly higher nitrogen uptake across all relative pressures (P/P_0_), consistent with its enhanced surface area and pore volume, as confirmed by BET and BJH analyses. The isotherm for AC-H_3_PO_4_ displays a pronounced Type H4 hysteresis loop, which is indicative of a complex pore network comprising both micropores and mesopores. The extension of this hysteresis loop to lower relative pressures suggests the presence of smaller mesopores and a broader pore size distribution, contributing to the material’s hierarchical porosity. In contrast, AC-H_2_SO_4_ exhibits a narrower Type H3 hysteresis loop, typically associated with slit-shaped pores or plate-like particles, which reflects a less complex pore structure.

The steeper slope of the AC-H_3_PO_4_ isotherm at low relative pressures (P/P_0_ < 0.1) indicates a higher degree of microporosity, aligning with the results of t-Plot analysis. Both carbons show gradual increases in nitrogen adsorption at medium to high relative pressures, confirming their mesoporous nature. However, AC-H_3_PO_4_ exhibits more substantial nitrogen uptake in this region, suggesting a larger mesopore volume compared to AC-H_2_SO_4_. These findings, in conjunction with BET and BJH analyses, demonstrate that activation with H_3_PO_4_ results in a more developed and hierarchical porous structure in AC-H_3_PO_4_. This enhanced porosity, coupled with a smaller particle size, suggests superior adsorption capacity and kinetics for a wide range of adsorbate molecules compared to AC-H_2_SO_4_. The observed hysteresis loops are a result of the physisorption of nitrogen gas under controlled conditions, without the use of an external desorbent. The adsorption and desorption processes occur at relative pressure ranges (P/P_0_), as indicated in the isotherms, and are consistent with the capillary condensation phenomenon within mesopores. This behavior is typical of mesoporous materials and further underscores the textural properties of the activated carbons derived from TG.

**Figure 7:**
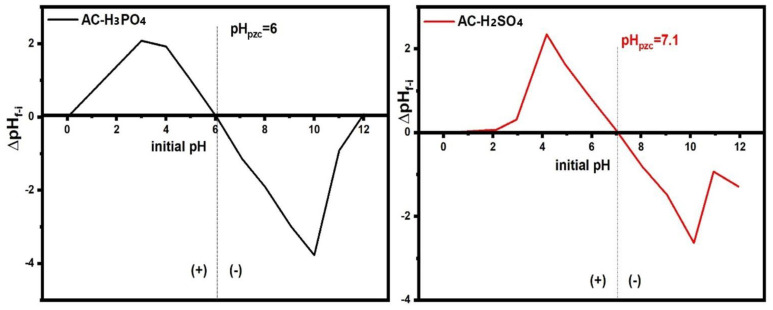
pHpzc graphical presentation of AC-H_3_PO_4_ and AC-H_2_SO_4_ samples.

pH_PZC_ analysis for ACs AC-H_3_PO_4_ and AC-H_2_SO_4_ (Figure 7) reveals significant differences in their surface charge properties and potential adsorption behavior, which are crucial for understanding their performance in pollutant removal. The pH_PZC_ of AC-H_3_PO_4_ was determined to be 6.0, indicating that its surface is positively charged at pH values below 6.0 and negatively charged at higher pH levels. In contrast, AC-H_2_SO_4_ displayed a higher pH_PZC_ of 7.1, suggesting a wider pH range in which it maintains a positive surface charge. These differences highlight the impact of the activation process on the surface chemistry of ACs. AC-H_2_SO_4_, activated with sulfuric acid, likely contains more basic functional groups, making it more effective in adsorbing anionic species. Conversely, AC-H_3_PO_4_, with its lower pH_PZC_, may better adsorb cationic pollutants in slightly acidic to neutral conditions. The pH-dependent adsorption efficiency of these ACs underscores the importance of selecting the appropriate pH conditions for optimizing pollutant removal in water treatment applications. The pronounced peak in the AC-H_2_SO_4_ curve further indicates stronger buffering capacity in acidic environments, which could enhance its applicability in such conditions. These findings underscore the critical role of activation methods in tailoring the surface properties of ACs for specific environmental applications.

### 3.2. Adsorption Performance and Analysis

#### 3.2.1. Kinetic Behaviors

**Figure 8:**
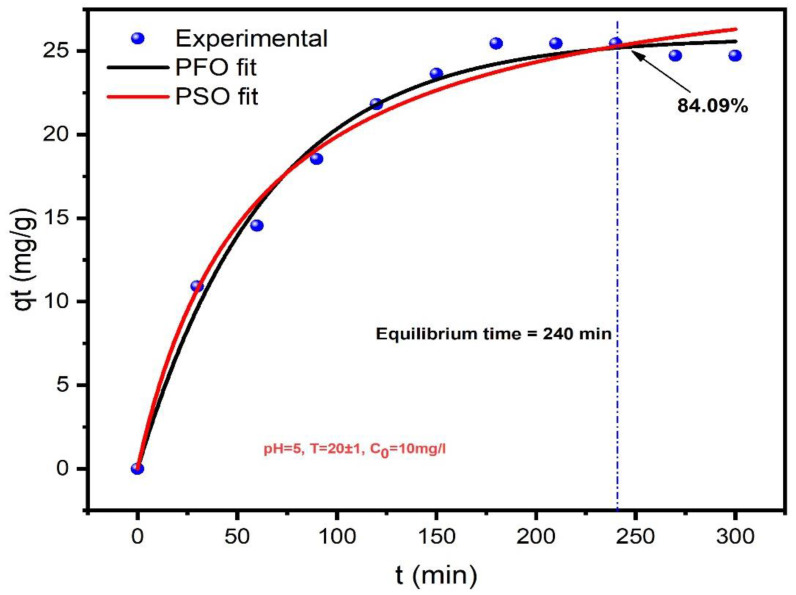
Kinetic presentation of GLY adsorption onto AC-H_3_PO_4_: experimental data, PFO and PSO models.

**Figure 9:**
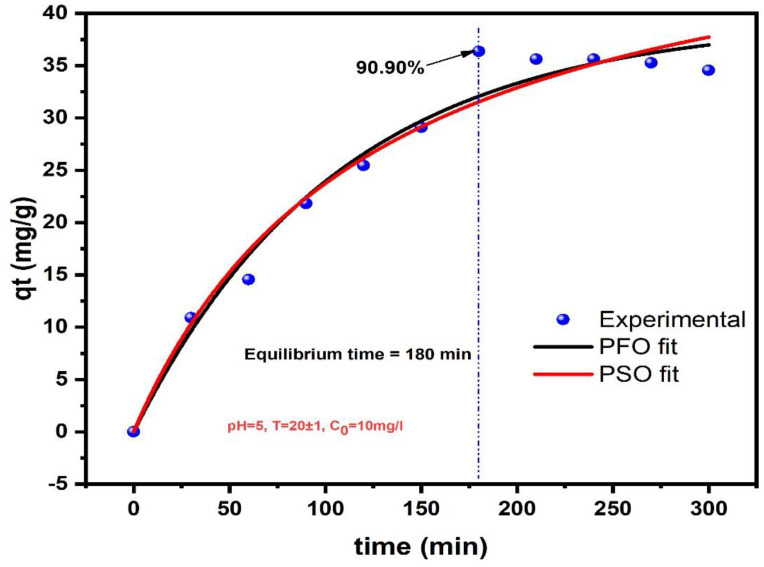
Kinetic presentation of GLY adsorption onto AC-H_2_SO_4_: experimental data, PFO and PSO models.

The kinetic analysis of GLY adsorption onto AC-H_3_PO_4_ and AC-H_2_SO_4_ provides valuable insights into the adsorption mechanisms and rate-controlling steps. Figure 8 and Figure 9 illustrate the time-dependent adsorption behavior, while Table 1 summarizes the kinetic parameters derived from PFO and PSO models. For AC-H_3_PO_4_, the PFO model demonstrated a slightly better fit (R^2^ = 0.989) compared to the PSO model (R^2^ = 0.983), with a lower χ^2^ value (0.77 vs. 1.244). Notably, the calculated q_e_ from the PFO model (25.817 mg/g) closely aligned with the experimental value, suggesting that the adsorption process may be primarily governed by physical interactions. In contrast, AC-H_2_SO_4_ exhibited a marginally better fit with the PFO model (R^2^ = 0.975) compared to the PSO model (R^2^ = 0.968), albeit with higher χ^2^ values for both models. The higher q_e_ value for AC-H_2_SO_4_ (39.338 mg/g) indicates its superior adsorption capacity, which can be attributed to its higher pHpzc (7.1) and potentially greater number of basic functional groups, as revealed by the characterization studies. The faster adsorption k_1_ observed for AC-H_3_PO_4_ (0.0155 min^−1^ vs. 9.4 × 10^−3^ min⁻¹ for AC-H_2_SO_4_) correlates with its larger surface area and more developed pore structure, facilitating rapid diffusion and adsorption of GLY molecules. These findings underscore the critical role of activation methods in tailoring the physicochemical properties of activated carbons for optimized adsorption performance.

#### 3.2.2. Isothermal Behaviors

**Figure 10:**
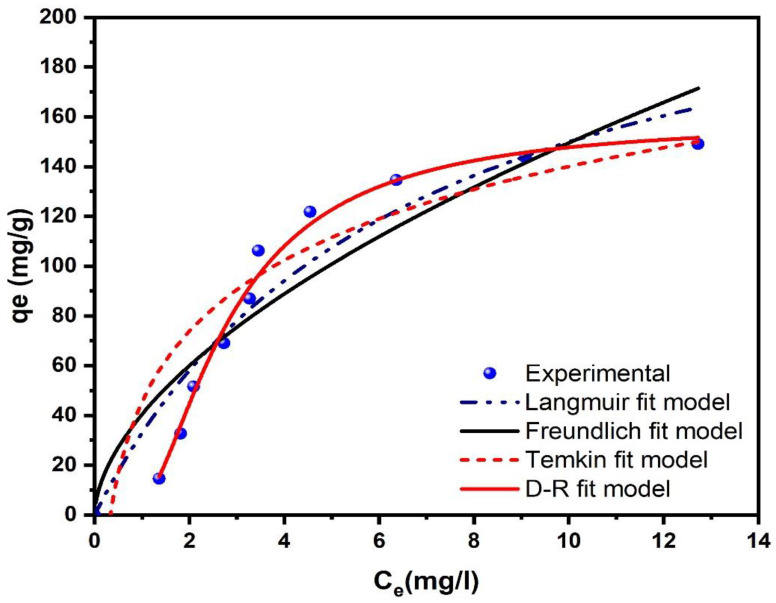
Isotherms presentation of GLY adsorption onto AC-H_3_PO_4_: experimental data and isotherm models.

**Figure 11:**
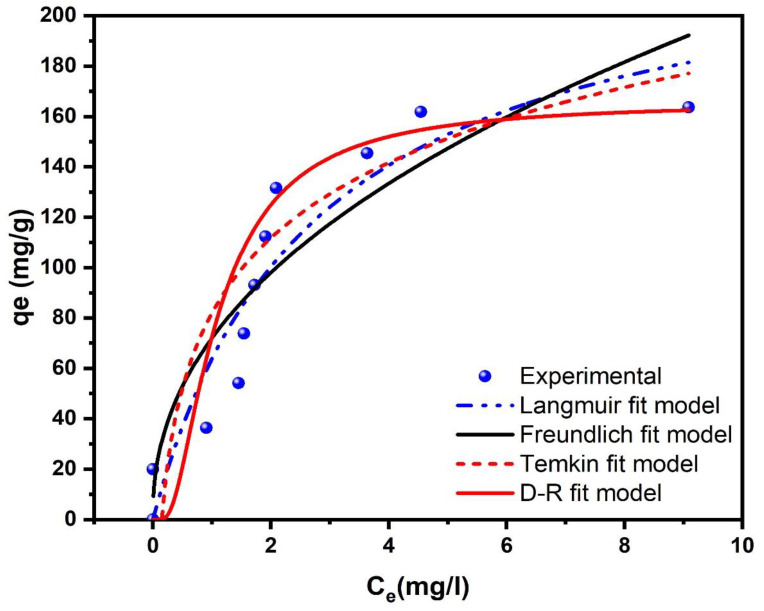
Isotherms presentation of GLY adsorption onto AC-H_2_SO_4_: experimental data and isotherm models.

The isotherm analysis of GLY adsorption onto AC-H_3_PO_4_ and AC-H_2_SO_4_ provides crucial insights into the adsorption mechanisms and surface interactions at equilibrium. Figure 10 and Figure 11 illustrate the equilibrium adsorption behavior, while Table 2 summarizes the parameters derived from Langmuir, Freundlich, Temkin, and D-R models. For AC-H_3_PO_4_, the D-R model demonstrated the best fit (R^2^ = 0.989, χ^2^ = 25.84), followed by Langmuir (R^2^ = 0.909, χ^2^ = 282.55), Temkin (R^2^ = 0.889, χ^2^ = 196.18), and Freundlich (R^2^ = 0.804, χ^2^ = 502.64) models. The superior fit of the D-R model suggests that the adsorption process is primarily governed by a pore-filling mechanism, which aligns with the highly developed porous structure of AC-H_3_PO_4_ observed in BET analysis (580.37 m^2^/g surface area). The calculated mean free energy of adsorption (E) from the D-R model (2.54 × 10^−2^ kJ/mol) indicates that physisorption is the dominant mechanism, corroborating the kinetic analysis findings.

Conversely, AC-H_2_SO_4_ exhibited a better fit with the Freundlich model (R^2^ = 0.974, χ^2^ = 50.32), followed by Temkin (R^2^ = 0.917, χ^2^ = 160.35), D-R (R^2^ = 0.903, χ^2^ = 171.56), and Langmuir (R^2^ = 0.893, χ^2^ = 394.19) models. The superior fit of the Freundlich model suggests a heterogeneous adsorption process with multilayer coverage, which is consistent with the more uniform surface topography and predominantly smaller pores observed in SEM analysis for AC-H_2_SO_4_. The Freundlich heterogeneity factor (n = 2.247) being greater than unity indicates favorable adsorption conditions. The Langmuir Qmax for AC-H_3_PO_4_ and AC-H_2_SO_4_ were 247.58 mg/g and 235.13 mg/g, respectively, reflecting the slightly higher adsorption potential of AC-H_3_PO_4_. This aligns with its larger surface area and more developed pore structure as revealed by BET analysis. However, the higher kL for AC-H_2_SO_4_ (0.372 vs. 0.153 for AC-H_3_PO_4_) suggests stronger adsorbate–adsorbent interactions, possibly due to its higher pHpzc and greater number of basic functional groups as indicated by the characterization studies. The Temkin model parameters provide insights into the heat of adsorption and adsorbent–adsorbate interactions. The higher Temkin constant A for AC-H_2_SO_4_ (6.66 L/mg vs. 3.01 L/mg for AC-H_3_PO_4_) suggests stronger binding energies, which correlates with its higher pHpzc and potentially greater electrostatic interactions with glyphosate molecules. The D-R model’s mean E for both adsorbents (2.54 × 10⁻^2^ kJ/mol for AC-H_3_PO_4_ and 4.11 × 10⁻^2^ kJ/mol for AC-H_2_SO_4_) falls within the range typically associated with physisorption processes (<8 kJ/mol), confirming that physical adsorption is the primary mechanism for glyphosate removal. These findings underscore the significant impact of activation methods on the physicochemical properties and adsorption behavior of activated carbons. The phosphoric acid activation of AC-H_3_PO_4_ resulted in a more heterogeneous surface with a wider range of pore sizes, favoring a pore-filling mechanism. In contrast, the sulfuric acid activation of AC-H_2_SO_4_ produced a more homogeneous surface with stronger adsorbate–adsorbent interactions, leading to multilayer adsorption. These differences in adsorption behavior can be directly attributed to the distinct surface chemistries and pore structures developed during the activation processes, as evidenced by the comprehensive characterization studies.

## 4. Conclusions

This study demonstrates the successful development of high-performance activated carbons from Tamarix gallica for glyphosate removal from aqueous solutions. The comparative analysis of phosphoric and sulfuric acid activation methods revealed distinct physicochemical properties and adsorption mechanisms. AC-H_3_PO_4_ exhibited superior surface area (580.37 m^2^/g) and pore development, favoring a pore-filling mechanism best described by the D-R model. In contrast, AC-H_2_SO_4_ showed stronger adsorbate–adsorbent interactions, resulting in multilayer adsorption fitting the Freundlich model. Both adsorbents demonstrated excellent glyphosate removal capacities, with AC-H_3_PO_4_ slightly outperforming AC-H_2_SO_4_ (Langmuir Qmax of 247.58 mg/g vs. 235.13 mg/g). Kinetic studies revealed that the PFO model best described the adsorption process for both adsorbents, with AC-H_3_PO_4_ exhibiting faster adsorption kinetics. The mean E values confirmed physisorption as the primary mechanism. These findings not only contribute to the growing body of knowledge on sustainable adsorbent materials but also offer practical insights for the design and optimization of water treatment processes targeting glyphosate contamination. This study underscores the importance of tailoring activation methods to enhance specific adsorption characteristics. Future research should focus on scaling up production, investigating regeneration methods, assessing the adsorbents’ performance in real-world water treatment scenarios, and exploring the potential for removing other emerging contaminants.

## Figures and Tables

**Table 1:** Kinetic parameters of GLY adsorption onto AC-H_3_PO_4_ and AC-H_2_SO_4_ surface.

Model	Parameters	AC-H_3_PO_4_	AC-H_2_SO_4_
PFO	q_e_ (mg/g)	25.817	39.338
	k_1_	0.0155	9.4 × 10^−3^
	R^2^	0.989	0.975
	χ^2^	0.77	4.213
PSO	q_e_ (mg/g)	31.37	53.49
	K_2_	5.52 × 10^−4^	1.492 × 10^−4^
	R^2^	0.983	0.968
	χ^2^	1.244	5.408

**Table 2:** Fitted models data for isotherm study of GLY adsorption onto AC-H_3_PO_4_ and AC-H_2_SO_4_ surface.

Model	Parameters	AC-H_3_PO_4_	AC-H_2_SO_4_
Langmuir	Qmax (mg/g)	247.58	235.13
	k_L_	0.153	0.372
	R^2^	0.909	0.893
	χ^2^	282.55	394.19
Freundlich	k_F_	40.42	71.95
	n	1.761	2.247
	R^2^	0.804	0.974
	χ^2^	502.64	50.32
Temkin	A (L/mg)	3.01	6.66
	b	59.39	56.66
	R^2^	0.889	0.917
	χ^2^	196.18	160.35
Dubinine–Radushkevish	q_m_ (mg/g)	158.43	165.54
	B_D_ (mol^2^K/j)	7.698	1.719
	E = 1/√(2B_D_) (KJ/mol)	2.54 × 10^−2^	4.11 × 10^−2^
	R^2^	0.989	0.903
	χ^2^	25.84	171.56

## Data Availability

The original contributions presented in this study are included in the article. Further inquiries can be directed to the corresponding author.
